# Metagenomic Analyses of Multiple Gut Datasets Revealed the Association of Phage Signatures in Colorectal Cancer

**DOI:** 10.3389/fcimb.2022.918010

**Published:** 2022-06-15

**Authors:** Wenxuan Zuo, Sonia Michail, Fengzhu Sun

**Affiliations:** ^1^ Quantitative and Computational Biology Department, University of Southern California, Los Angeles, CA, United States; ^2^ Department of Pediatrics, Keck School of Medicine of the University of Southern California, Los Angeles, CA, United States

**Keywords:** gut virome, metagenomics, colorectal cancer, virus-host association, CRC prediction

## Abstract

The association of colorectal cancer (CRC) and the human gut microbiome dysbiosis has been the focus of several studies in the past. Many bacterial taxa have been shown to have differential abundance among CRC patients compared to healthy controls. However, the relationship between CRC and non-bacterial gut microbiome such as the gut virome is under-studied and not well understood. In this study we conducted a comprehensive analysis of the association of viral abundances with CRC using metagenomic shotgun sequencing data of 462 CRC subjects and 449 healthy controls from 7 studies performed in 8 different countries. Despite the high heterogeneity, our results showed that the virome alpha diversity was consistently higher in CRC patients than in healthy controls (p-value <0.001). This finding is in sharp contrast to previous reports of low alpha diversity of prokaryotes in CRC compared to healthy controls. In addition to the previously known association of *Podoviridae*, *Siphoviridae* and *Myoviridae* with CRC, we further demonstrate that *Herelleviridae*, a newly constructed viral family, is significantly depleted in CRC subjects. Our interkingdom association analysis reveals a less intertwined correlation between the gut virome and bacteriome in CRC compared to healthy controls. Furthermore, we show that the viral abundance profiles can be used to accurately predict CRC disease status (AUROC >0.8) in both within-study and cross-study settings. The combination of training sets resulted in rather generalized and accurate prediction models. Our study clearly shows that subjects with colorectal cancer harbor a distinct human gut virome profile which may have an important role in this disease.

## Introduction

Colorectal cancer (CRC) is the third most common cancer worldwide causing at least 500,000 deaths annually (Bray et al., 2018). Most colorectal cancers are caused by complex genetic and environmental factors. While only a small proportion of cases are explained by genetic mutations (Toma et al., 2012), over 70% of them are due to environmental and lifestyle factors (Frank et al., 2017). Potential CRC risk factors include older age, lack of physical activity, diet rich in red meat, smoking and alcohol use (Fund and for Cancer Research, 2007; Watson and Collins, 2011).

The human gut microbiome, the microbial communities inhabiting our gastrointestinal tract, can greatly influence human health through the immune and metabolic systems (Holmes et al., 2012). Many studies have demonstrated the complicated associations between gut microbiome and human diseases such as inflammatory bowel disease (IBD) (Norman et al., 2015; Zuo et al., 2022), metabolic diseases (Qin et al., 2012) and autoimmune diseases (Tomofuji et al., 2022). The human gut microbiome, which could be altered by some of the mentioned risk factors, has also been considered as one of the most important environmental factors in the development of CRC. Indeed, several studies have already shown the structural alterations of gut microbiome among CRC patients (Thomas et al., 2019; Wirbel et al., 2019).

Most of the current microbiome studies concentrate on prokaryotes as they constitute most of the genetic materials in the gut microbial community. On the other hand, the number of viruses that infect bacteria referred to as bacteriophages or simply phages for short outnumber the bacteria by tenfold. These bacteriophages impact the microbial community directly through their own genes or indirectly by infecting their hosts. However, the importance of the gut virome was vastly understudied due to the relatively low fraction of viral genetic materials in microbiomes, despite their larger numbers compared to that of prokaryotes. The lack of well-curated viral reference genomes has also hampered efforts to accurately study the virome. To overcome these issues, experimental techniques to enrich virus-like particles (VLP) in microbiome studies have been developed (Ludwig and Wagner, 2007). Through the enrichment of VLPs, it has been shown that several viral taxa were associated with some diseases such as ulcerative colitis (UC) (Zuo et al., 2019), a subtype of IBD. However, only a very limited number of VLP studies related to CRC have been conducted thus far.

Until recently, most studies used NCBI virus database as references for virome studies. However, the fraction of viruses in NCBI only represented a tiny fraction of all the viruses. To overcome this issue, metagenome assembled genomes (MAG) were constructed from a large number of metagenomes and computational virus identification algorithms such as VirFinder (Ren et al., 2017) and/or VirSorter (Roux et al., 2015). This allowed the identification of more viruses resulting in larger virus databases. Gregory et al. (2020) constructed the Gut Virome Database (GVD) from 2,697 public metagenomic samples, Paez-Espino et al. (2019) built the IMG/VR database based on stool samples from the Human Microbiome Project (HMP) and Camarillo-Guerrero et al. (2021) formed the Gut Phage Database (GPD) from 28,060 metagenomes. These large virome databases provided new resources for investigating the relationship between viruses and complex diseases. However, to the best of our knowledge, no studies have been carried out to investigate the relationship between human gut virome and CRC using such newly developed databases.

In this study, we analyzed metagenomic shotgun sequencing data from 7 CRC studies, including 462 CRC subjects and 449 healthy controls. We evaluated changes in the gut virome of CRC subjects and the heterogeneity among different studies by obtaining the virus abundance profiles and mapping the reads against the Gut Phage Database (Camarillo-Guerrero et al., 2021), the largest phage database available to date. Secondly, we performed differential abundance analysis to identify CRC-associated viral species, bacterial species and metabolic pathways. Next, phage-bacterium associations were identified to illustrate the symbiotic network in CRC. In addition, we estimated the diagnostic ability of the viral profiling by the performance of classification models trained on either within-study and cross-study settings. Finally, we measured the generalizability and robustness of the classifiers by pooling these datasets together. Our results provide comprehensive insight into the links between gut virome and colorectal cancer.

## Materials and Methods

### Cohort Description

We collected 911 healthy control and CRC subjects from 7 publicly available datasets from 7 countries and 3 continents (**Table 1** and **Supplementary Tables S1, S2**). All 7 studies used fecal shotgun sequencing to compare the gut microbiome of CRC patients to that of healthy controls. Fecal samples from all participants of these studies were collected before treatment, thus excluding the cancer treatment as a potential confounding factor. Characteristics of these datasets are shown in **Table 1**. Sequencing depth distributions within these datasets are shown in **Supplementary Figure S1**.

**Table 1:** Characteristics of metagenomic datasets used in this study.

Study	No. of controls	No. of CRC	Country	Reference
Zeller	93	91	France/Germany	(Zeller et al., 2014)
Yu	54	74	China	(Yu et al., 2017)
Feng	63	46	Austria	(Feng et al., 2015)
Vogtmann	52	52	USA	(Vogtmann et al., 2016)
Thomas	52	61	Italy	(Thomas et al., 2019)
Yachida	40	40	Japan	(Yachida et al., 2019)
Yang	95	98	China	(Yang et al., 2020)
**Total**	**449**	**462**		

### Quantification of Viral Abundance

Centrifuge v1.0.4 (Kim et al., 2016) with default parameters was used to map reads from each sample against the Gut Phage Database (GPD) (Camarillo-Guerrero et al., 2021), since Centrifuge yielded more accurate estimation of relative abundance at species and genus rank (Meyer et al., 2022). GPD is a recently published database containing 142,809 non-redundant gut phage genomes. On average, 31.84% reads of each sample were mapped to GPD. Although a small proportion (< 10%) of viral reads could still be mapped to NCBI bacterial reference genomes, it does not substantially affect the statistical results. Such reads are potentially caused by the prophage in the bactieral reference genomes or the non-viral genome in GPD (the allowed false positive rate of GPD was 0.25%). The distribution of viral mapping rates within each dataset is shown in **Supplementary Figure S2**. The number of unique mappings given by Centrifuge was further normalized with trimmed mean of M values (TMM) (Robinson et al., 2010) using the edgeR package (Robinson et al., 2010) to obtain the TMM normalized abundance profiling. Although other viruses such as eukaryotic viruses and endogenous retroviruses are also important components of gut virome, their mapping rate was low (< 0.02%). Therefore, we focused our analyses on gut phages in this study.

### Taxonomic Annotation

A protein-level comparison was used for the species-level annotation. First, open reading frames (ORFs) in viral genomes from GPD were predicted using Prodigal meta (v2.6.3) (Hyatt et al., 2010). The predicted ORFs in the viral genomes were searched against the RefSeq protein database (downloaded in December 2021, containing 577,484 proteins) using DIAMOND blastp (v2.0.13) (Buchfink et al., 2021) with e-value less than . Each ORF was assigned to the protein with the highest bit score. Each viral genome was assigned taxonomy based on the majority of taxa within that genome using a voting system for virus taxonomic assignment at different taxon levels (Minot et al., 2013; Hannigan et al., 2015; Zuo et al., 2019). Viral genomes with less than two ORFs were considered unclassified viral species (Tomofuji et al., 2022).

In summary, 134,871 viral genomes in GPD were annotated with species or higher-level taxon, respectively. Camarillo-Guerrero et al. (2021) also used HMMER (Eddy, 1998) to query each protein sequence within the viral genome against the ViPhOG database for taxonomic annotation. Only 16,636 viral genomes in GPD were annotated with family-level taxon. Out of these 16,636 viral genomes, we assigned family-level taxon to 15,603 genomes with 70.7%(11,033) having the same predicted taxon as obtained by Camarillo-Guerrero et al. (2021).

### Viral Functional Profiling

In order to study the viral gene expression in CRC patients, we used viral reads (reads that were mapped to GPD) to obtain the gene family and pathway profiling. HUMAnN3 (Beghini et al., 2021) along with its ChocoPhlAn pangenome database and UniRef90 EC filtered database was used to predict the Pfam protein domains and Gene Ontology terms, with the reported abundance value shown as RPKs (reads per kilobases). In total, 462 pathways and 497,967 ( ± 132,909) were identified.

### Viral Diversity, Multivariate Analysis and Meta-Analysis

Shannon index (Spellerberg and Fedor, 2003), Heip evenness (Heip, 1974) and Chao1 richness (Chao, 1984) were used to measure viral diversity for each dataset at species, genus and family levels, respectively. To study the association between disease status and alpha diversity as well as the impact of age, gender and body mass index (BMI in kg/) on this association (Thomas et al., 2019), we performed a naive linear model (*Y*∼*Disease*) and an age-, gender- and BMI-adjusted linear model (*Y*∼*Disease*+*Age*+*Gender*+*BMI*), where *Y* is the log-transformed alpha diversity. Coefficients and p-values of the disease variable were obtained to perform the comparisons. Meta-analysis was implemented using the R package metafor (Viechtbauer, 2010). Standardized mean difference of alpha diversity was calculated for each taxonomic level to obtain the random effect model. Heterogeneity among studies was quantified by (percentage of total heterogeneity on total variability) and the p-value was obtained by Cochran’s Q test (Cochran, 1950).

### Principal Coordinate Analysis

The dissimilarity between CRC cases and healthy controls in all 7 datasets were measured by principal coordinate analysis (PCoA) (Dray et al., 2006) based on Bray-Curtis distance (Bray and Curtis, 1957). PCoA was performed on either combined datasets or each separate dataset. Permutational multivariate analysis of variance (PERMANOVA) (Anderson, 2001) was used to quantify the heterogeneity among different datasets and the separation between healthy controls and CRC subjects.

### Differential Abundance Analysis

To identify viral species, genus, families, as well as the viral functions that are differentially abundant in the CRC group, we used DESeq2 (Love et al., 2014) to perform differential analysis based on their abundance profiling. Taxa with low variance (less than half of the median) or not found in ≥90% samples were removed (Lloyd-Price et al., 2019).

The exact test in DESeq2 was used to calculate the p-value for each variable. Multiple hypothesis tests were adjusted using the Benjamini-Hochberg false discovery rate (BH-FDR) procedure (Benjamini and Hochberg, 1995). Associations with FDR less than 0.05 were considered significant.

### Quantification of Bacterial Abundance and Virus-Bacterium Association

Centrifuge v1.0.4 (Kim et al., 2016) with default parameters was used to map non-viral reads from each sample against the UHGG database (Almeida et al., 2021) to obtain the bacterial abundance profile. The TMM normalized abundance profiles were obtained in the same way as the viral abundance profile.

We chose the 27 bacterial species that are differentially abundant between CRC cases and healthy controls in all 7 datasets for further analysis. Spearman’s correlation coefficients based on the bacterial species abundance and viral family abundance were calculated. Fisher’s z-transformation (Fisher, 1915) and meta-analysis were used to derive a random effect model on Spearman’s correlation coefficients. The p-values were adjusted by the BH procedure. Only associations with adjusted p-value <0.05 were considered significant.

### Random Forest Classifier for Within-Study and Cross-Study Prediction

We used six types of microbiome quantitative profiles: GPD genome-level, taxonomic family-level, genus-level, species-level TMM normalized abundance estimated by Centrifuge (Kim et al., 2016) and gene-family and pathway abundance (in RPKs) estimated by HUMAnN3 (Beghini et al., 2021), to predict CRC status using random forests.

Since the random forests algorithm (Breiman, 2001) has been proven to have better performance than other machine learning models, especially on microbial abundance data (Pasolli et al., 2016; Gao et al., 2022), all experiments were carried out using the random forests classifier implemented by the python package scikit-learn v1.0.2 (Pedregosa et al., 2011). We set the number of estimators as 1000 (Thomas et al., 2019; Gao et al., 2022) and all other parameters as their default values (Probst et al., 2019) in all prediction experiments. The area under the ROC curve (AUROC) was used as a criterion measuring the performance of every prediction model.

To accurately measure the performance, generalizability and robustness of predictions models, we performed within-dataset, cross-dataset and leave-one-dataset-out (LODO) predictions (Thomas et al., 2019). The within-dataset analysis was performed by repeating 10-fold cross validation 20 times (Thomas et al., 2019). The average AUROC based on 200 runs was calculated as the final measurement.

The cross-dataset analysis was performed by pairwise datasets prediction. For each pair of all 7 datasets, one was used as the training set, and the other one as the validation set. This step was also repeated 20 times to reduce randomness within the algorithm.

The LODO analysis consisted of the validation set as one of the 7 datasets and the training set as the pooled samples from the other 6 datasets. This approach substantially increased the size of the training set, which could potentially improve the performance and generalization of the random forests classifier. It also helped reduce profiling differences between different data batches.

## Results

### Case-Control Comparison Showed Higher Viral Diversity in CRC Samples Compared to Healthy Controls

**Figure 1:**
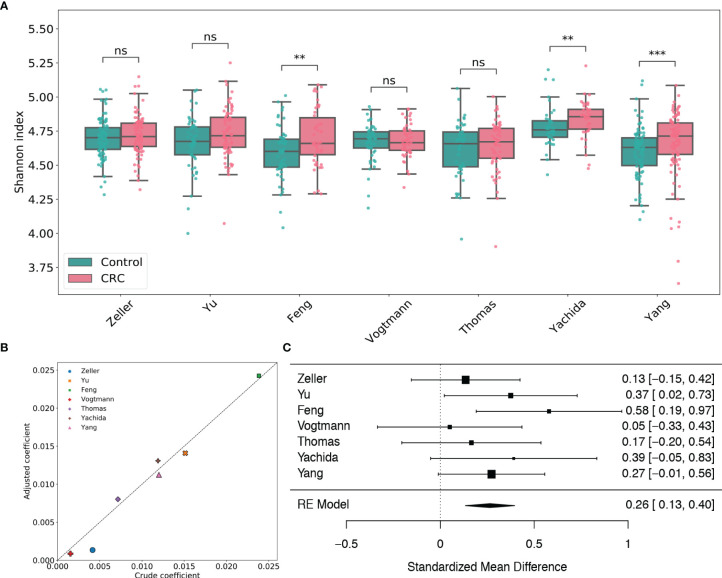
Analysis of viral species Shannon diversity within each dataset. **(A)** Boxplots of viral species-level Shannon index for gut samples of CRC subjects and healthy controls stratified by disease status in each dataset. BH adjusted p-values were calculated using the two-tailed Wilcoxon rank-sum test. ns: p> 0.05, *p< 0.05, **p< 0.01, ***p< 0.001. **(B)** Multivariate analysis of the adjusted impact of age, gender and BMI on Shannon diversity. **(C)** Forest plot showing effect sizes from a meta-analysis on species-level diversity. RE Model: Random effect model.

By taxonomic annotation, GPD genomes were assigned to 2,056 viral species, 673 viral genera, 24 viral families and 7 viral orders, respectively. To study the gut viral structural alteration within each dataset, we performed case-control comparison for species-level, genus-level and family-level alpha diversity [measured by Shannon index (Spellerberg and Fedor, 2003), Heip evenness (Heip, 1974) and Chao1 richness (Chao, 1984)] for each dataset. **Figure 1A** and **Supplementary Figures S3, 4** showed that the virome alpha diversity and evenness are higher in CRC subjects than healthy controls at species, genus and family levels, which are consistent with the findings in Nakatsu et al. (2018). The increments are significant in the Feng, Yachida and Yang datasets (adjusted p-values of two-tailed Wilcoxon rank-sum test are less than 0.01). This positive association between species viral diversity and CRC can be further observed in the multivariate analysis (**Figure 1B** and **Supplementary Table S3**), since all coefficients given by linear models are positive. Potential confounding factors such as age, gender and BMI do not meaningfully impact the contribution of disease status to the alpha diversity. It should be noted that although the viral alpha diversity in CRC cases is not statistically different from that in healthy controls, they are in the same direction as the other three datasets. Results of meta-analysis (**Figure 1**) also showed this significant positive association (μ=0.26, p-value<0.0001) with no heterogeneity observed in alpha diversity (*I* ^2^=0.0%, Q test p-value =0.49). Similarly, the evenness also exhibited a positive association with CRC (μ=0.28, p-value<0.0001) without significant heterogeneity (*I* ^2^=0.0%, Q test p-value =0.89, **Supplementary Figure S4**). Whereas, the Chao1 richness (**Supplementary Figure S5**) indicated either negative associations or positive associations with CRC in different studies. However, the random effect model obtained by the meta-analysis suggested a positive effect size (μ=0.12, p-value=0.318) of the richness, although this association was not significant. Altogether, these findings highlighted associations between dysbiosis of gut virome and CRC.

### Principal Coordinates Analysis Among Different Studies

**Figure 2:**
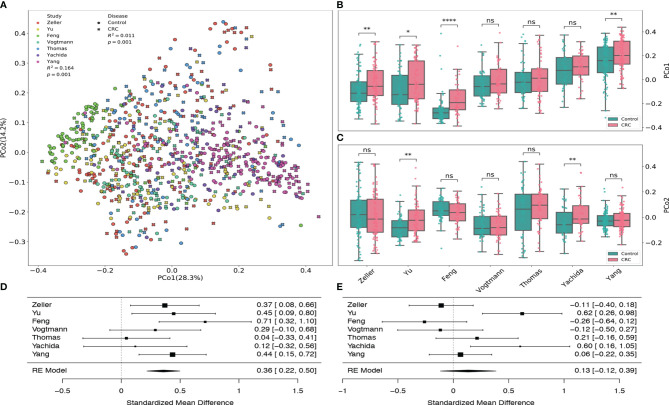
Principal coordinates analysis of all samples based on Bray–Curtis distance. **(A)** PCoA plot of gut samples of CRC subjects and healthy controls in each dataset. *R* ^2^ values and p-values were calculated by PERMANOVA. **(B)** Boxplots of the first principal coordinates (PCo1) in each dataset. **(C)** Boxplots of the second principal coordinates (PCo2) in each dataset. BH adjusted p-values were calculated using the two-tailed Wilcoxon rank-sum test. ns:p> 0.05, *p< 0.05, **p< 0.01, ***p< 0.001, ****p< 0.0001. **(D)** Forest plot showing effect sizes from a meta-analysis on PCo1. **(E)** Forest plot showing effect sizes from a meta-analysis on PCo2. RE Model: Random effect model.

By using the principal coordinates analysis based on Bray-Curtis distance, we assessed both the heterogeneity among different datasets from various studies and the dissimilarity in the gut viral communities between healthy controls and CRC subjects. **Figure 2A** revealed that the heterogeneity among 7 datasets had significant effect (PERMANOVA *R* ^2^=0.164, p-value=0.001) on the gut viral composition, which is consistent with the results from Thomas et al. (2019) and Wirbel et al. (2019).

Samples from Feng dataset (light green), Zeller dataset (red) and Yang dataset (fuchsia) tend to cluster to the left, middle and right among all subjects, respectively. Boxplots (**Figures 2B, D**) also show that their first principal coordinates (explained 28.3% of the variance) are significantly greater in the CRC group compared to the control group, suggesting the existence of within-study clusters. The significance of these separations is validated by the PEMANOVA between the viral dissimilarity and disease status (*R* ^2^=0.011, p-value=0.001). Meta-analysis also indicates substantial heterogeneity on the second principal coordinates (**Figures 2C, E**, μ = 0.13, p-value = 0.29, *I* ^2^=71.88%, Q test p-value = 0.0027). In addition, **Supplementary Figure S6** further demonstrates significant separations between healthy controls and CRC subjects within each dataset. All PERMANOVA p-values are less than 0.05 except the p-value of the Vogtmann dataset.

### Differential Abundance Analysis Revealed Important Viral Taxon and Metabolic Pathways Associated With CRC

**Figure 3:**
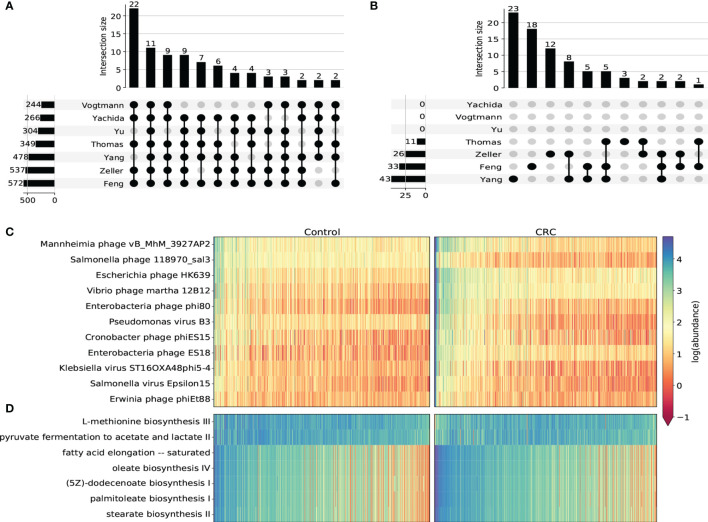
Differential abundance analysis on taxonomic and functional viral profiles. **(A)** UpSet plot showing the number of shared differentially abundant viral species determined by species-level TMM normalized abundance and DESeq2. Only viral species differentiated in at least 5 datasets were displayed. **(B)** UpSet plot showing the number of shared differentially abundant viral pathways determined by HUMAnN3 pathway abundance and DESeq2. **(C)** Heatmap showing the log transformed TMM normalized abundance of viral species differentiated in all 7 datasets. **(D)** Heatmap showing the log transformed HUMAnN3 pathway abundance of pathways differentiated in at least 3 datasets.

We next used DESeq2 to perform differential abundance analysis on species-level, genus-level, family-level and pathway abundance (**Supplementary Tables S4–S7**). In general, most taxa identified in this analysis were from the *Caudovirales* order. Most of them were enriched in CRC cohorts compared to healthy controls. We found 11 CRC-enriched viral species (p-value <10^-5^, **Figures 3A, C** and **Supplementary Figure S7**) from 3 phage families that were significantly enriched in CRC cohorts in all 7 datasets, including *Erwinia phage phiEt88*, *Klebsiella virus ST16OXA48phi5-4*, *Vibrio phage martha 12B12*, *Mannheimia phage vB_MhM_3927AP2*, *Salmonella phage 118970_sal3* from *Myoviridae*, *Salmonella virus Epsilon15* from *Podoviridae* and *Pseudomonas virus B3*, *Escherichia phage HK639*, *Enterobacteria phage phi80*, *Enterobacteria phage ES18*, *Cronobacter phage phiES15* from *Siphoviridae*. Among these species, *Klebsiella virus* (Canizalez-Roman et al., 2022) *Enterobacteria phage phi80* and *Salmonella phage* (Gao et al., 2021) were found to increase in the CRC group. *Erwinia phage* and *Vibrio phage* were also reported to contribute to CRC progression (Nakatsu et al., 2018; Ng et al., 2019). All viral genera that were significant in at least 6 datasets were found to be increased in the CRC group (**Supplementary Figure S8**). In regard to family-level taxon, besides *Myoviridae*, *Podoviridae* and *Siphoviridae* that were obtained from the species-level analysis, we additionally identified *Drexlerviridae*, *Inoviridae* and *Herelleviridae* that were significantly associated with CRC (**Supplementary Figure S9**). While all other 5 families were found to be more abundant in the CRC group, *Herelleviridae*, a recently established phage family in the *Caudovirales* order (Barylski et al., 2020), was observed to be significantly depleted in the CRC group for most datasets (**Supplementary Figure S9C**). Phages in the *Herelleviridae* family typically infect members of the *Firmicutes* phylum (Barylski et al., 2020) and serve as a potential treatment to the infection of intestinal epithelium-like environment (Núñez-Sánchez et al., 2020).

Viral functional signatures were described by the differential abundance analysis of KEGG pathways. Even though most gut virome functions remain to be uncurated with only a small portion of viral reads that can be functionally characterized, we detected 7 metabolic pathways (p-value <10^-5^) that were notably associated with CRC. Similar to taxonomic taxa, most pathways were enriched in the CRC groups in most datasets (**Figures 3B, D** and **Supplementary Figure S10**), including stearate biosynthesis II, oleate biosynthesis IV, fatty acid elongation – saturated, palmitoleate biosynthesis I and (5Z)-dodecenoate biosynthesis I. These pathways were substantially associated with fatty acid biosynthesis such as stearic acid, oleic acid and palmitoleic acid, all of which were demonstrated to modulate the metabolic profiles and increase the risk of CRC (Chen et al., 2016; Serini et al., 2018; Kim, 2019). On the contrary, we discovered two pathways that were significantly decreased in the CRC groups. One is L-methionine biosynthesis iii, which is an intracellular regulator that functions to inhibit the proliferation of colorectal cancer cells (Módis et al., 2014). The other depleted pathway is pyruvate fermentation to acetate and lactate ii. This pathway ferments fiber into acetate, which may play an important role in the turnover of the colonic epithelium to maintain the normal homeostasis (Eslami et al., 2020). Therefore, the inactivity of these two pathways may serve as future targeted therapies of CRC.

Taken together, the differential abundance analysis based on the taxonomic and functional profiling indicated that the structural alteration of gut viruses, mainly bacteriophages, was substantial in CRC cohorts. Although most bacteriophages were enriched in CRC groups and consequently caused more active expression of fatty acid biosynthesis, some were observed to decrease in the CRC groups potentially inactivating the protective inhibition process of immune regulation. These results can further expand our understanding of the potential contribution of the gut virome in CRC.

### Interkingdom Association Between Viral Families and Bacterial Species

**Figure 4:**
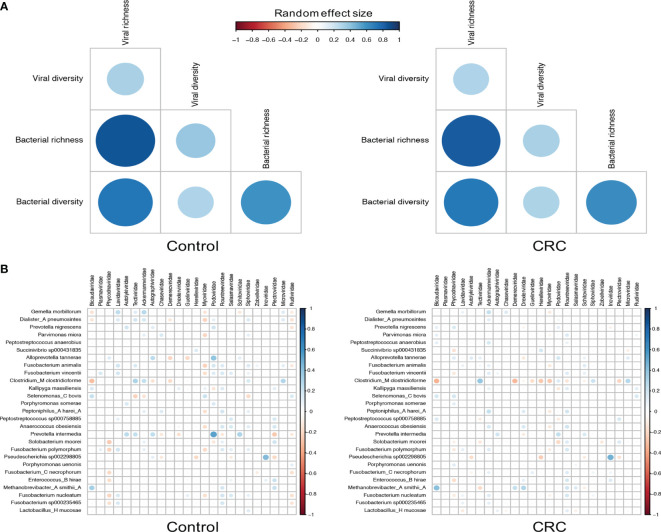
Correlations between viral families and bacterial species. **(A)** Random effect size of Spearman’s correlation coefficients between the diversity and richness of bacteria and viruses in healthy controls and CRC subjects. Correlations with BH adjusted p-values <0.05 are displayed. **(B)** Random effect size of Spearman’s correlation coefficients between the abundance of all 24 viral families and that of 27 differentially abundant bacterial species. Correlations with BH adjusted p-values <0.05 are displayed. The size and color of circles indicate the extent of correlation.

Since most viruses in human gut are bacteriophages that either lyse their hosts or alter their functions, we then characterized the relationship between bacteriophages and their hosts by assessing the correlation between their abundance and alpha diversity (Norman et al., 2015; Zuo et al., 2019). To identify differentially abundant bacterial species, we performed differential analysis on each dataset (**Supplementary Table S8**) and found 27 bacterial species significant in all 7 datasets. Although the bacterial richness (**Supplementary Figures S11B, D**) did not show consistent differences among datasets (meta-analysis *I* ^2^=81.81%, Q test p-value <0.0001), we did find that the bacterial alpha diversity substantially decreased in CRC in most datasets (**Supplementary Figures S11A, C**, meta-analysis *I* ^2^=73.31%, Q test p-value =0.0029), which may have been the result of the expansion of the viral community. **Figure 4A** showed a positive bacterium-virus correlation in terms of diversity and richness in both control and CRC groups. While the direction of the correlation between diversity and richness within a kingdom remains the same in the control and CRC groups, the positive interkingdom association was weaker in the CRC group, especially for the association between viral alpha diversity and bacterial richness.

The decrements of the association between bacteria and viruses in CRC could be further quantified by the correlation between viral families and bacterial species. **Figure 4B** showed similar patterns of virus-bacterium associations in both control and CRC groups. Positive correlations included *Bicaudaviridae* and *Methanobrevibacter smithii*, *Tectiviridae* and *Clostridium_M clostridioforme*, and negative correlations with *Bicaudaviridae* and *Clostridium_M clostridioforme*, *Myoviridae* and *Gemella morbillorum*. In the CRC group, however, the positive correlations between *Podoviridae* and several bacterial species as well as the negative correlations between *Myoviridae* and most bacterial species were markedly decreased. Altogether, these results revealed a complex alteration of virus-bacterium relationship in CRC. The reduction in these correlations implies a shrinkage of symbiotic network and highlights the importance of virus-bacterium equilibrium in the maintenance of intestinal stability.

### Random Forests Classifiers Accurately Predict CRC Status Based on Human Gut Virome Profiles

**Figure 5:**
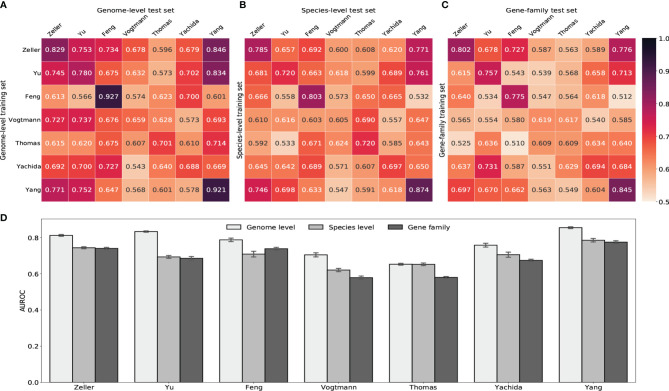
Prediction performances of random forest classifiers based on gut viral abundance. **(A)** Within and cross study AUROC matrix obtained by using GPD genome-level abundance. The diagonal refers to results of cross validation within each dataset. Off-diagonal values refer to prediction results trained on the study of each row and tested on the study of each column. **(B)** Within and cross study AUROC matrix obtained by using species-level abundance. See **Supplementary Figures S12A, B** for genus-level and family-level AUROC. **(C)** Within and cross study AUROC matrix obtained by using gene-family abundance. See **Supplementary Figure S12C** for pathway AUROC. **(D)** LODO results with the x axis indicating the study left out as the validation set and other studies combined as the training set.

We next built random forest models using either gut viral taxonomic profiling or viral functional profiling to distinguish between healthy controls and CRC subjects. Despite the ethnic difference and the heterogeneity of sequencing techniques, classifiers achieved high AUROC with gut viral profiles in both within-study and cross-study predictions. **Figure 5** showed that AUROC scores of within-study cross validation range between 0.65 and 0.92 on GPD genome-level abundance. Classification models had weaker performance in the Vogtmann and Thomas datasets. Compared with the AUROC scores of within-study prediction, the AUROC scores of cross-study slightly dropped. The highest decrease came from the models trained on the Feng and Yang datasets, which indicates weaker generalization of these two datasets. The overfitting within the Feng dataset was also observed in a whole gut microbiome study by Wirbel et al. (2019). The performances of random forest classifiers using other taxonomic levels such as species, genus and family abundance (**Figures 5B, C** and **Supplementary Figure S12**) were lower than that based on genome-level abundance, suggesting the loss of viral signature when tracing upward the taxonomic tree. However, gene-family abundance with more functional units (more than 400,000) did not necessarily enhance the overall performance of the random forest model, reflecting the redundant nature of the information provided by viral functional profiles.

Increasing the size of training set generally improves the prediction ability of machine learning models. Therefore, we further estimated the diagnostic ability of the random forest classifier by the leave-one-dataset-out validation (LODO) (Thomas et al., 2019; Wirbel et al., 2019). The LODO results of GPD genome-level models again outperformed all other models trained on other type of abundance (**Figure 5D** and **Supplementary Figure S12D**). The LODO results of GPD genome-level models had a stable AUROC range from 0.75 to 0.85, with the Vogtmann and the Thomas datasets regarded as outliers. The lower AUROC in the Vogtmann dataset suggested that the long-time (>25 years) freezing before sequencing altered the viral community structure in fecal samples (Vogtmann et al., 2016; Wirbel et al., 2019). In addition, the weak prediction result of the Thomas dataset was potentially due to the relatively shallow sequencing depth compared to other datasets (**Supplementary Figure S1**).

Bacterial signatures of the human gut have been shown to be predictive of CRC status (Thomas et al., 2019; Wirbel et al., 2019; Gao et al., 2022). To study whether the viral signatures can further enhance the prediction performance of this disease, we combined both bacterial and viral abundance profiles together and re-run the random forest model. The results are shown in **Supplementary Figure S13**, which does not show a better performance when this combination is used. The bacterial abundance profile itself shows a high AUROC (>0.8). The combination of bacterial and viral species abundance profiles did not increase the AUC scores compared to the bacterial abundance profile alone. Moreover, adding the viral genome abundance can even reduce the prediction performance of the random forest model using bacterial abundance profile. This is due to the much higher dimension of viral genome abundance, which mitigates the effect of bacterial signatures. One potential explanation for this observation is that the human gut virome does not independently contribute to CRC development, but rather interacts with the prokaryotes to impact CRC, resulting in high correlation between the gut viral abundance profile and bacterial abundance profile.

On the whole, the LODO analysis revealed the random forest models trained on these heterogeneous datasets have solid generalization and robustness to make accurate predictions on other metagenomic CRC studies. The prediction ability (AUROC >0.80) achieved based on gut viral signatures was competitive with that of whole gut microbial signatures (AUROC >0.83) (Thomas et al., 2019; Wirbel et al., 2019; Gao et al., 2022). Although it can not further enhance the performance of the bacterial signatures, the prediction reuslts still show the important role of viruses in the homeostasis of gut microbiota.

## Discussion

Analysis of the composition of the gut microbiome provides new insight in the understanding of the etiology and pathophysiology of many gastrointestinal diseases. The development of colorectal cancer is complex and involves genetic and environment factors such as the gut microbiome (Frank et al., 2017). Despite the fact that many studies have demonstrated specific microbial signatures in CRC, much remains to be explored in the structure of the gut virome. To our knowledge, this study is the most comprehensive analysis of the association between gut virome and CRC using the largest collection of datasets to date. Although there is technical heterogeneity among different datasets, we found some consistent patterns and prediction abilities among these datasets, including the viral diversity, CRC-associated viral species, metabolic pathways as well as robust and accurate diagnostic models.

The alpha diversity of gut viruses was found to be much higher in the CRC cohorts at the species, genus and family levels. Combined with results of previous studies (Coker et al., 2019; Cheng et al., 2020), we demonstrate that the dysbiosis of the intestinal microbiota is highly associated with CRC, perhaps the enrichment of viral species results in more lytic infections in the host, thus significantly depleting the gut bacterial organisms and prompting the development of CRC. In addition to the alpha diversity, the principal coordinate analyses with Bray-Curtis distance and the PERMANOVA test further unraveled the separation between healthy controls and CRC subjects.

The presence of 11 viral species and 10 viral genera was commonly associated with CRC in the majority of the 7 datasets employed in this study. At the family level, *Myoviridae*, *Podoviridae*, *Siphoviridae*, *Drexlerviridae* from *Caudovirales* order and *Inoviridae* from *Tubulavirales* order were increased in the CRC groups. Among these enriched families, *Myoviridae*, *Podoviridae* and *Siphoviridae* were frequently reported to be associated with CRC (Hannigan et al., 2018; Sánchez-Alcoholado et al., 2020) and other human diseases such as IBD (Clooney et al., 2019) and autoimmune diseases (Tomofuji et al., 2022), which substantially validate our results. We also discovered that the viral family *Herelleviridae* significantly was significantly depleted in CRC groups. As a relatively new viral family (Barylski et al., 2020), *Herelleviridae* contains phage species that may have therapeutic potential for gastrointestinal infections (Núñez-Sánchez et al., 2020). Phages in the Herelleviridae family typically infect members of the Firmicutes phylum (Barylski et al., 2020) and have been shown to display Ig-like domains on their virions that contribute to the integrity and heath of intestinal barrier function serving as a potential treatment targeting the intestinal epithelium (Núñez-Sánchez et al., 2020). Therefore, the depletion of this family may lead to intestinal epithelial dysregulation permissive to the development of tumors. Moreover, several metabolic pathways were identified in subjects with CRC in this study. Five metabolic pathways related to fatty acid biosynthesis were found to be more active in CRC. These pathways have been shown to increase risk of CRC in prior studies (Chen et al., 2016; Serini et al., 2018; Kim, 2019). Furthermore, two other pathways namely L-methionine biosynthesis iii and pyruvate fermentation to acetate and lactate ii were inactive in CRC. pathways Both have been evinced to be firmly linked to inhibiting the proliferation of tumors (Módis et al., 2014; Eslami et al., 2020). Such pathways may serve as potential therapy targets in the future.

Our correlation analysis further reveals the interkingdom association in CRC. Although the alpha diversity demonstrates a positive correlation between viral families and bacterial species in both healthy controls and CRC, the reciprocity between them considerably weakened in CRC, especially in the network between *Podoviridae*, *Myoviridae* and most displayed bacterial species. These relationships are important in the virus-bacterium interaction and their effect on the intestinal health.

Finally, the diagnostic models we built based on the viral abundance and random forest algorithm outperformed all other prior studies of the gut virome (Nakatsu et al., 2018; Gao et al., 2021). Despite the high performance of distinguishing CRC being achieved with the whole gut microbiome (Thomas et al., 2019; Wirbel et al., 2019), our virome-based classifiers had competitive results in both within-study and cross-study validations. Remarkably, the LODO experiment showed that the diagnostic models are quite robust, which suggests that the combination of heterogeneous datasets can substantially improve the sensitivity and accuracy for detecting CRC cases in other independent datasets.

In conclusion, we performed a comprehensive gut virome case-control study, revealing the significant contribution of the gut virome in CRC. The detected gut virobiota, which links the virome and bacteriome as combined diagnostic models, unveil a new perspective of the gut virome in the pathogenesis of CRC.

## Data Availability Statement

The original contributions presented in the study are included in the article/**Supplementary Material**. Further inquiries can be directed to the corresponding author.

## Authors Contributions

FS designed and supervised the study. WZ did all analyses and visualization. WZ drafted the manuscript. SM provided clinical implications of the results. SM and FS polished the manuscript and all authors contributed to finalize the manuscript. All authors read and approved the final version of the manuscript.

## Funding

This work was partially supported by the National Institutes of Health [NIH Grants R01GM120624 and 1R01GM131407].

## Conflict of Interest

The authors declare that the research was conducted in the absence of any commercial or financial relationships that could be construed as a potential conflict of interest.

## Publisher’s Note

All claims expressed in this article are solely those of the authors and do not necessarily represent those of their affiliated organizations, or those of the publisher, the editors and the reviewers. Any product that may be evaluated in this article, or claim that may be made by its manufacturer, is not guaranteed or endorsed by the publisher.
