# Supplementary material for: Metagenomic Analyses of Multiple Gut Datasets Revealed the Association of Phage Signatures in Colorectal Cancer
Source: Front Cell Infect Microbiol. 2022 Jun 15;12:918010. doi: 10.3389/fcimb.2022.918010 (PMC9240273; doi:10.3389/fcimb.2022.918010)
Supplement: Supplementary file 1 [file DataSheet_1.pdf]

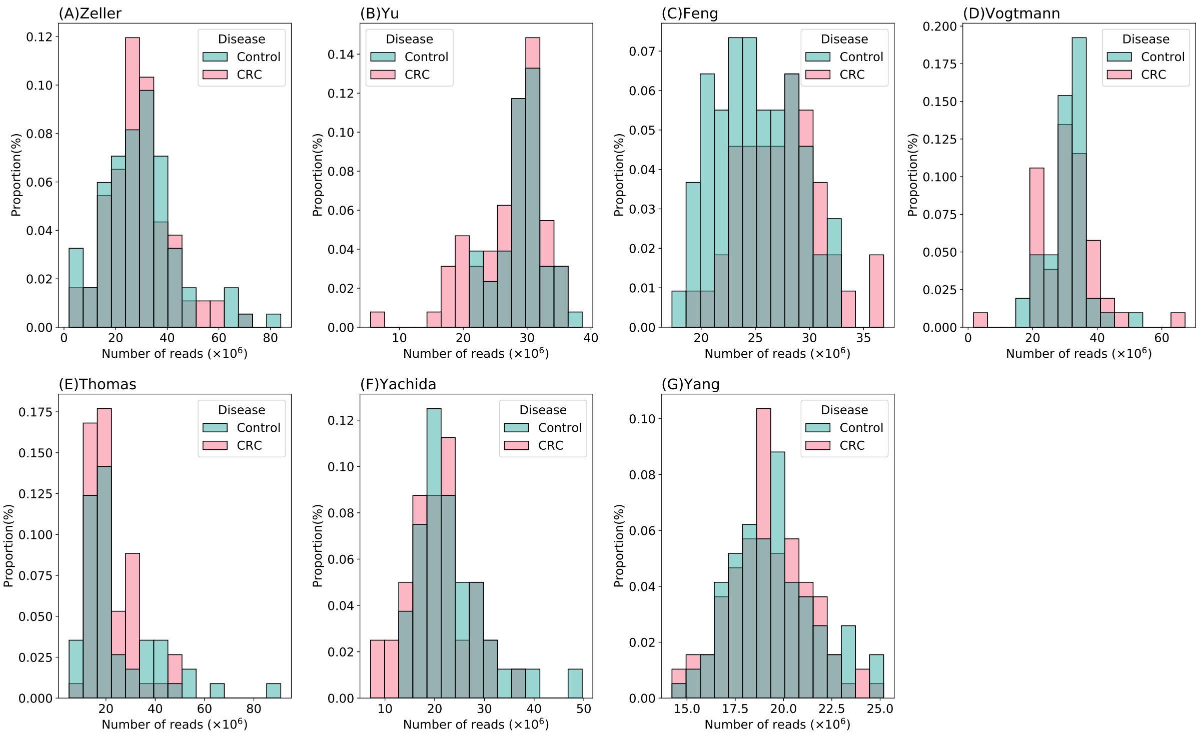


Figure S1: The distribution of sequence depths within each dataset used in this study.

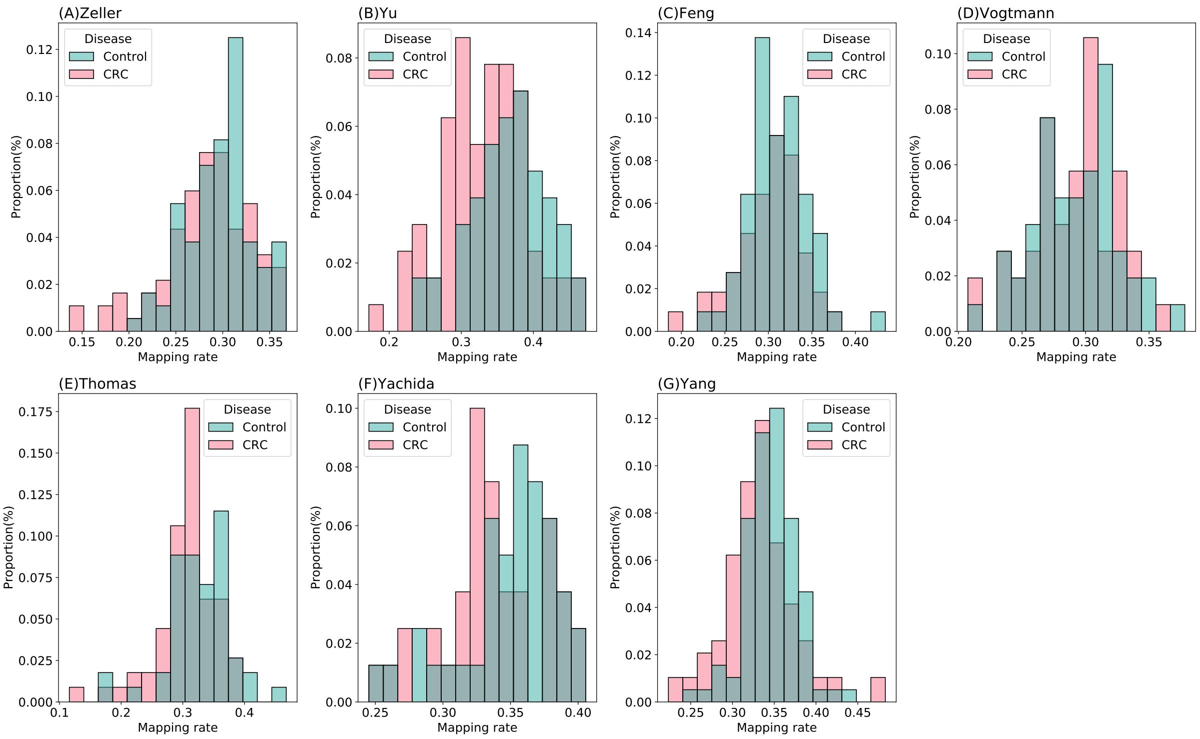


Figure S2: The distribution of mapping rates for each dataset used in this study.

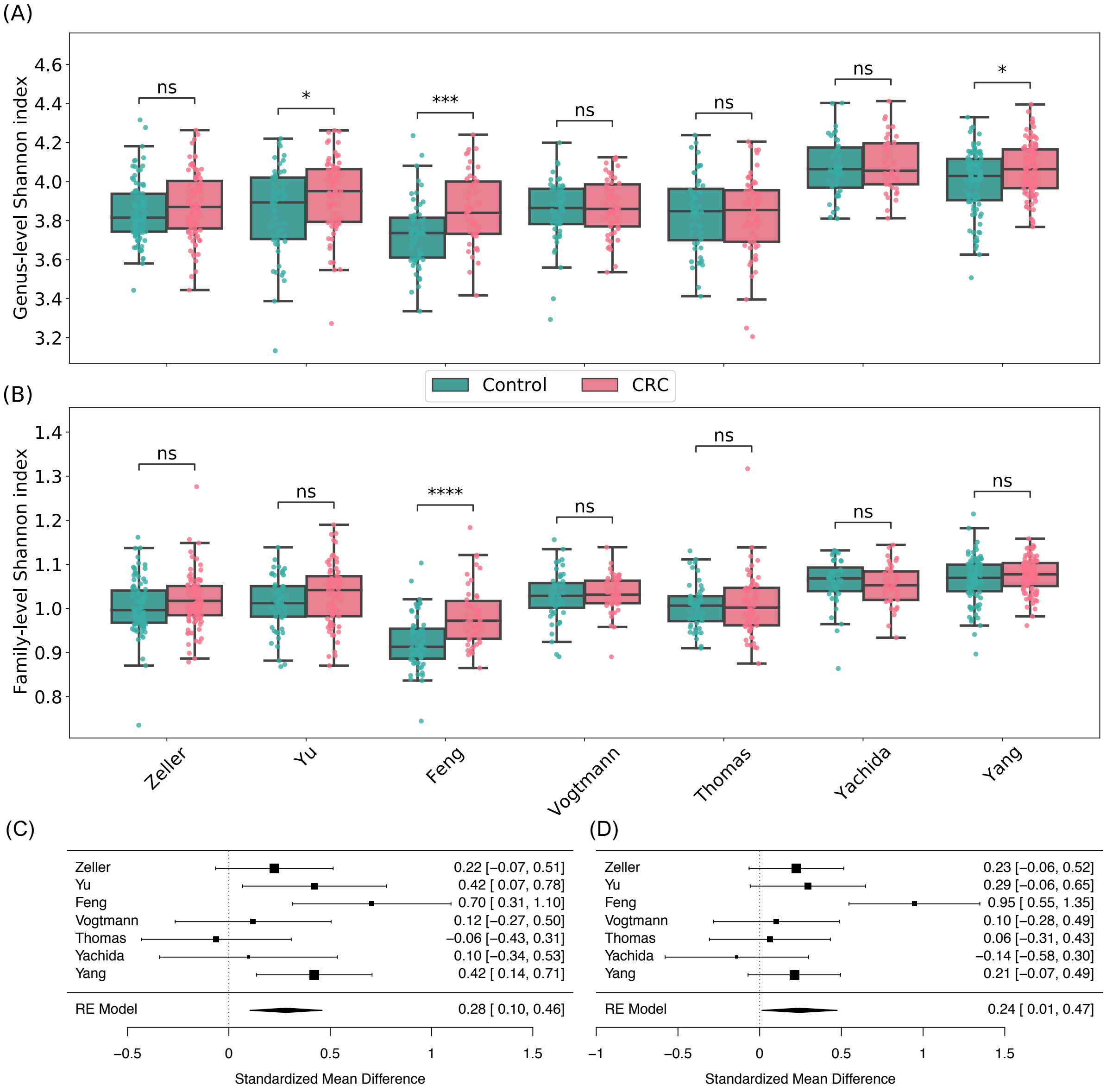


Figure S3: Boxplots of (**A**) genus-level and (**B**) family-level Shannon diversity within each dataset. P-values were calculated using the two-tailed Wilcoxon rank-sum test. ns:p$>$ 0.05, *p$<$ 0.05, **p$<$ 0.01, ***p$<$ 0.001, ****p$<$ 0.0001. (**C**) Forest plot showing effect sizes from a meta-analysis on genus-level diversity ($I^2 = 44.10\%$, Q test p-value$=$ 0.094). (**D**) Forest plot showing effect sizes from a meta-analysis on family-level diversity ($I^2 = 66.51\%$, Q test p-value$=$ 0.012). RE Model: Random effect model.

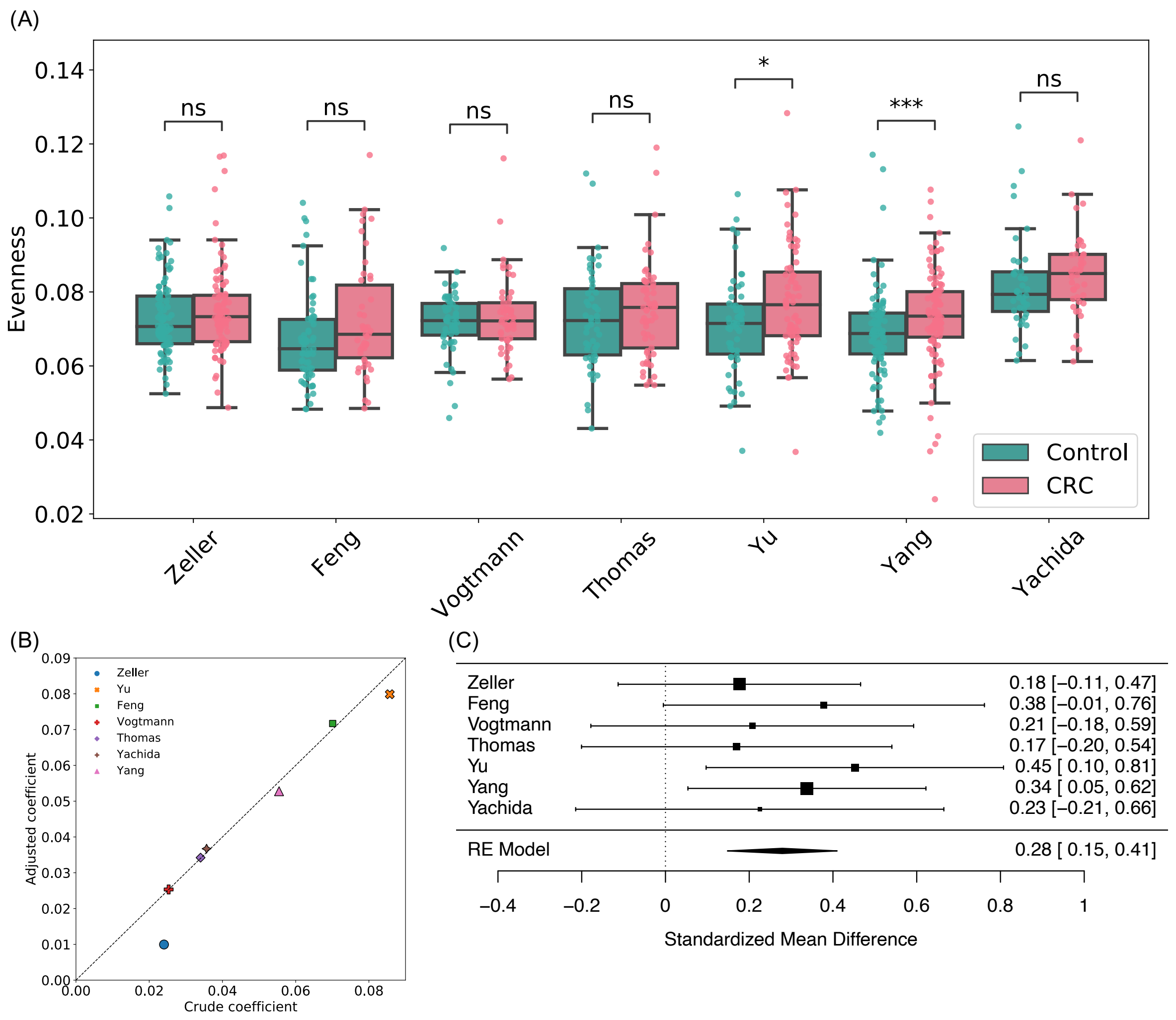


Figure S4: Analysis of viral species Heip evenness within each dataset. (**A**) Boxplots of viral species-level Heip evenness for gut samples of CRC subjects and healthy controls stratified by disease status in each dataset. BH adjusted p-values were calculated using the two-tailed Wilcoxon rank-sum test. ns:p$>0.05$, *p$<0.05$, **p$<0.01$, ***p$<0.001$. (**B**) Multivariate analysis of the adjusted impact of age, gender and BMI on Heip evenness. (**C**) Forest plot showing effect sizes from a meta-analysis on species-level evenness ($I^2 = 0.00\%$, Q test p-value$= 0.89$). RE Model: Random effect model.

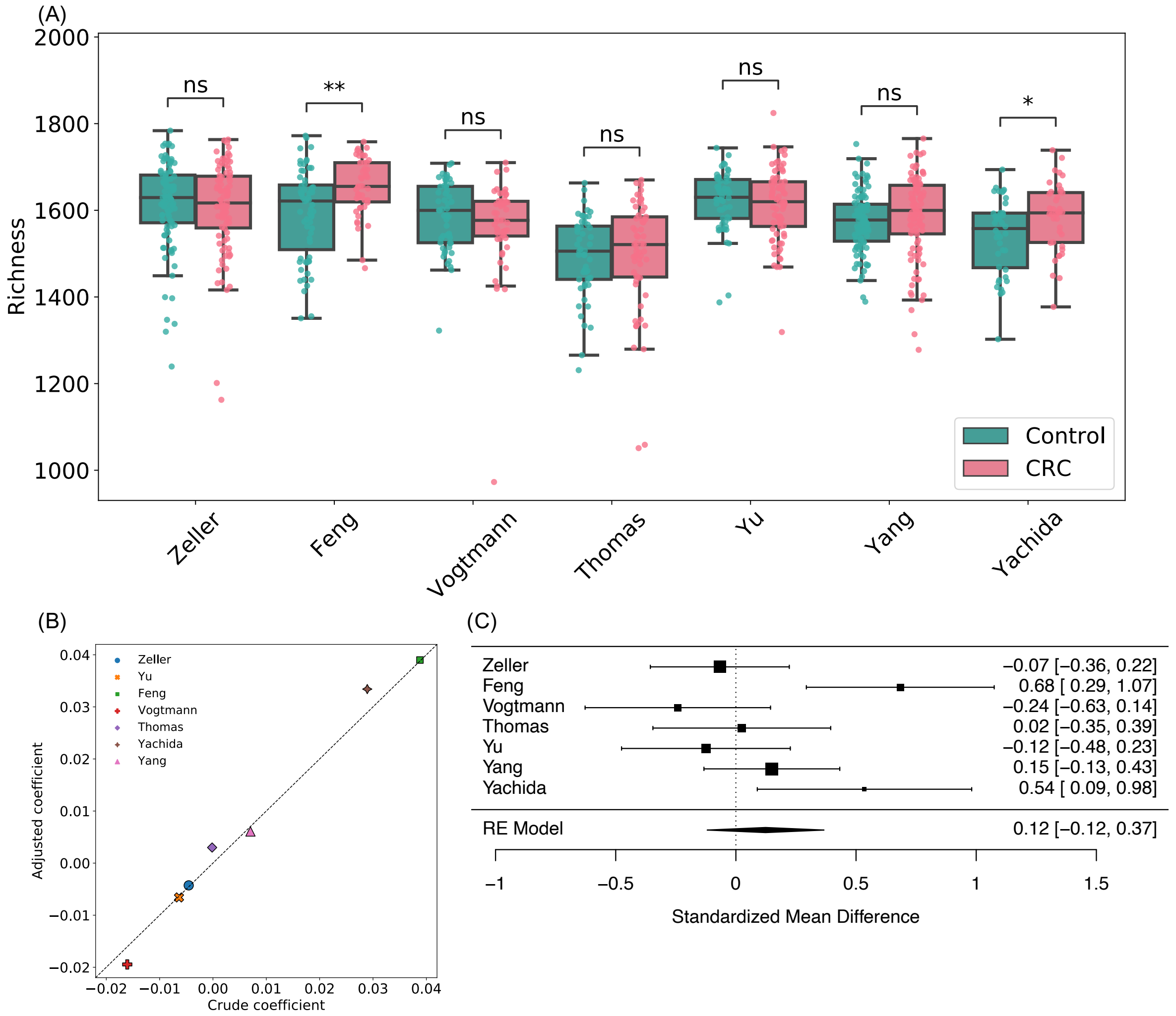


Figure S5: Analysis of viral species Chao1 richness within each dataset. (**A**) Boxplots of viral species-levelChao1 richness for gut samples of CRC subjects and healthy controls stratified by disease status in each dataset. BH adjusted p-values were calculated using the two-tailed Wilcoxon rank-sum test. ns:p$>0.05$, *p$<0.05$, **p$<0.01$, ***p$<0.001$. (**B**) Multivariate analysis of the adjusted impact of age, gender and BMI on Chao1 richness. (**C**) Forest plot showing effect sizes from a meta-analysis on species-level richness ($I^2 = 69.93\%$, Q test p-value$=0.005$). RE Model: Random effect model.

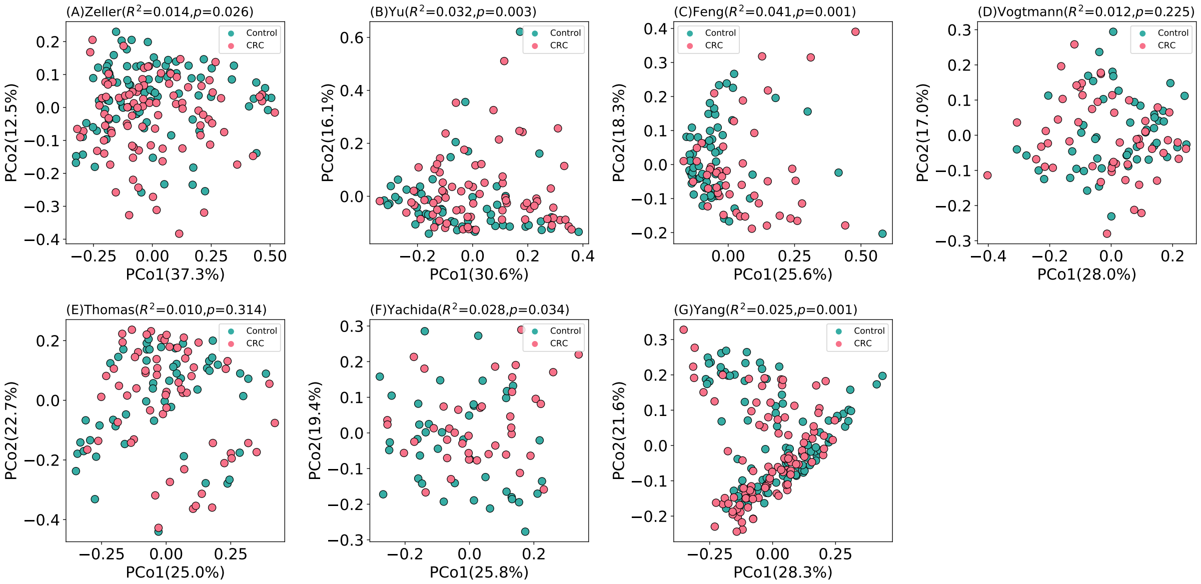


Figure S6: PCoA plot of gut samples of CRC subjects and healthy controls for each separate dataset. $R^2$ values and p-values on subtitles were calculated by PERMANOVA to quantify the separation of the samples into two groups.

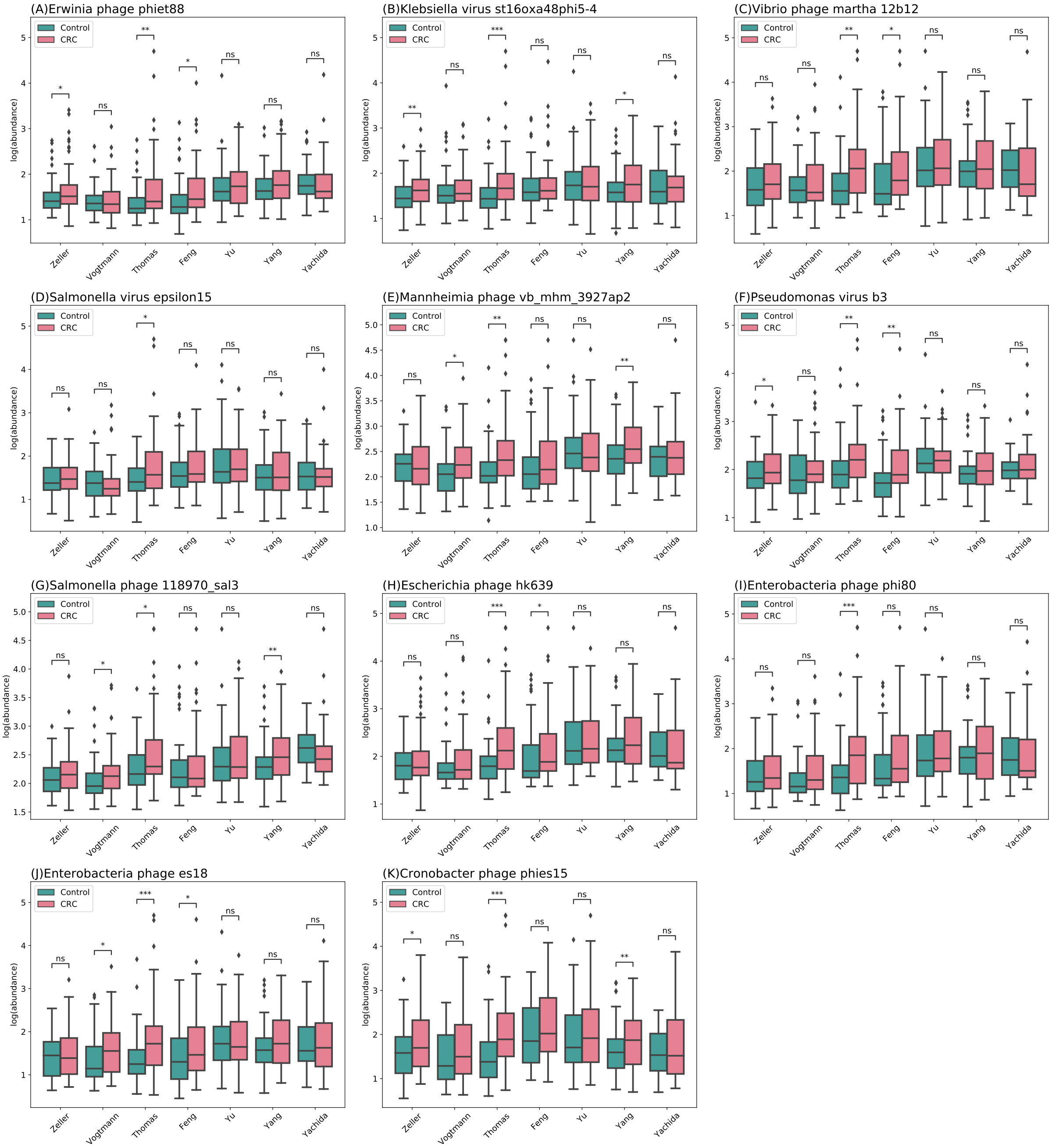


Figure S7: Boxplots showing the log transformed TMM normalized abundance of significant CRC-associated viral species within each dataset. P-values were calculated using the two-tailed Wilcoxon rank-sum test. ns:p$>0.05$, *p$<0.05$, **p$<0.01$, ***p$<0.001$, ****p$<0.0001$.

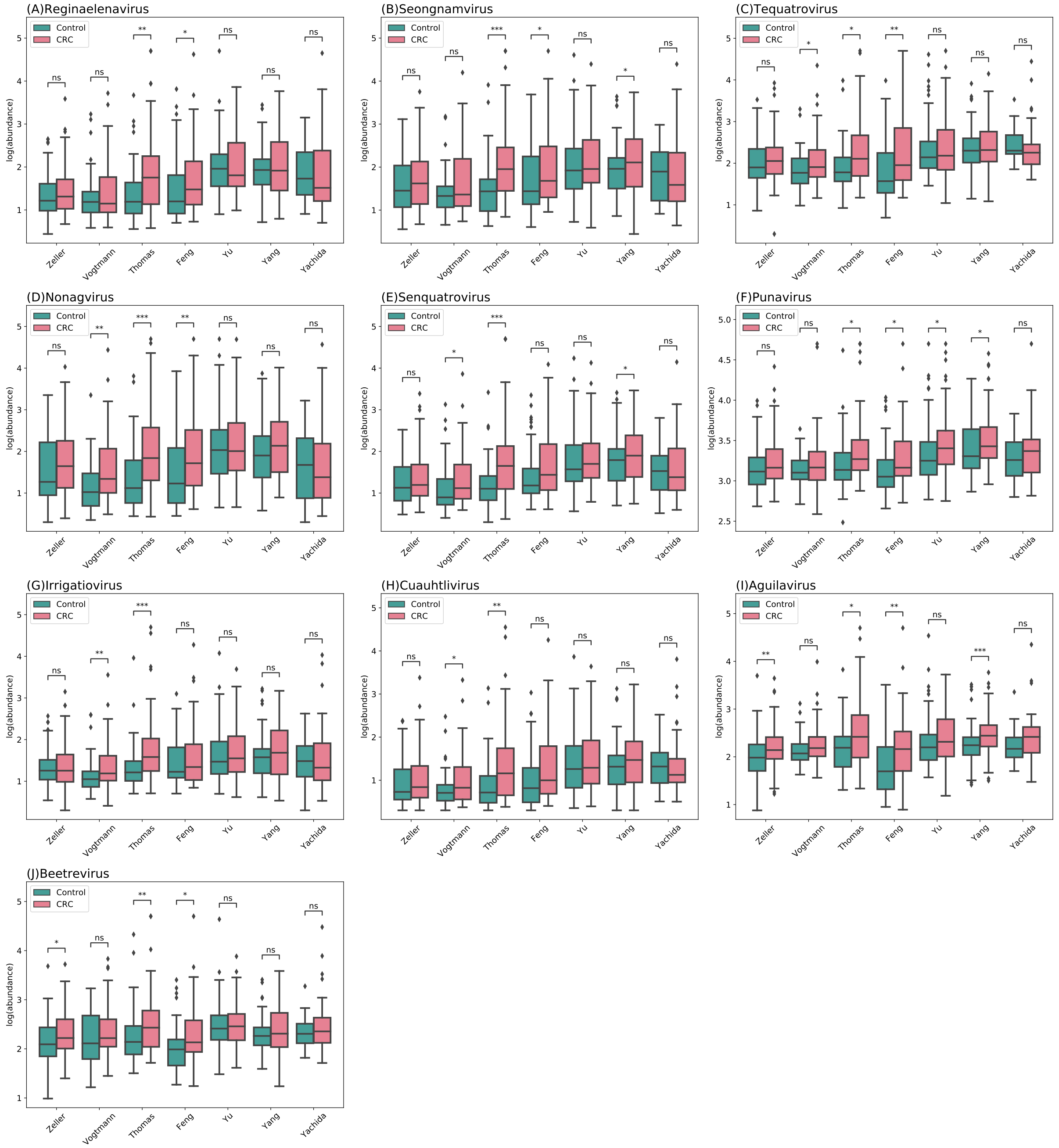


Figure S8: Boxplots showing the log transformed TMM normalized abundance of significant CRC-associated viral genera within each dataset. P-values were calculated using the two-tailed Wilcoxon rank-sum test. ns:p$>0.05$, *p$<0.05$, **p$<0.01$, ***p$<0.001$, ****p$<0.0001$.

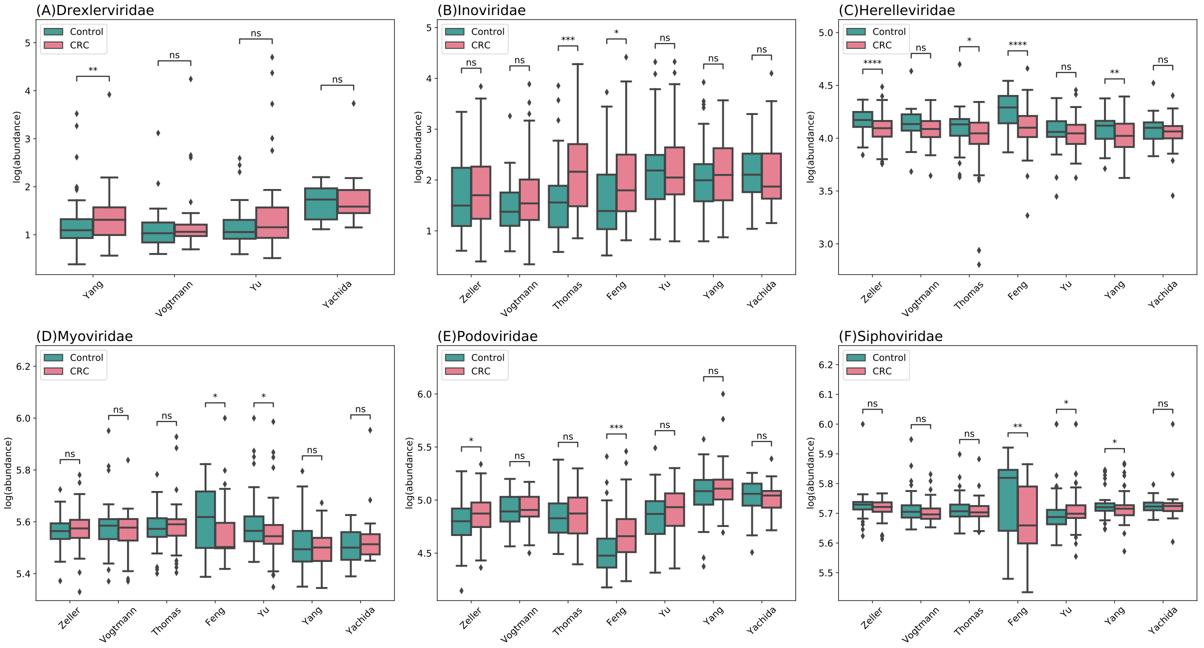


Figure S9: Boxplots showing the log transformed TMM normalized abundance of significant CRC-associated viral families ((**A**) Drexlerviridae, (**B**) Inoviridae, (**C**) Herelleviridae)) and families to which significant CRC-associated viral species belong ((**D**) Myoviridae, (**E**) Podoviridae, (**F**) Siphoviridae)) within each dataset. P-values were calculated using the two-tailed Wilcoxon rank-sum test. ns:p$>$ 0.05, *p$<$ 0.05, **p$<$ 0.01, ***p$<$ 0.001, ****p$<$ 0.0001.

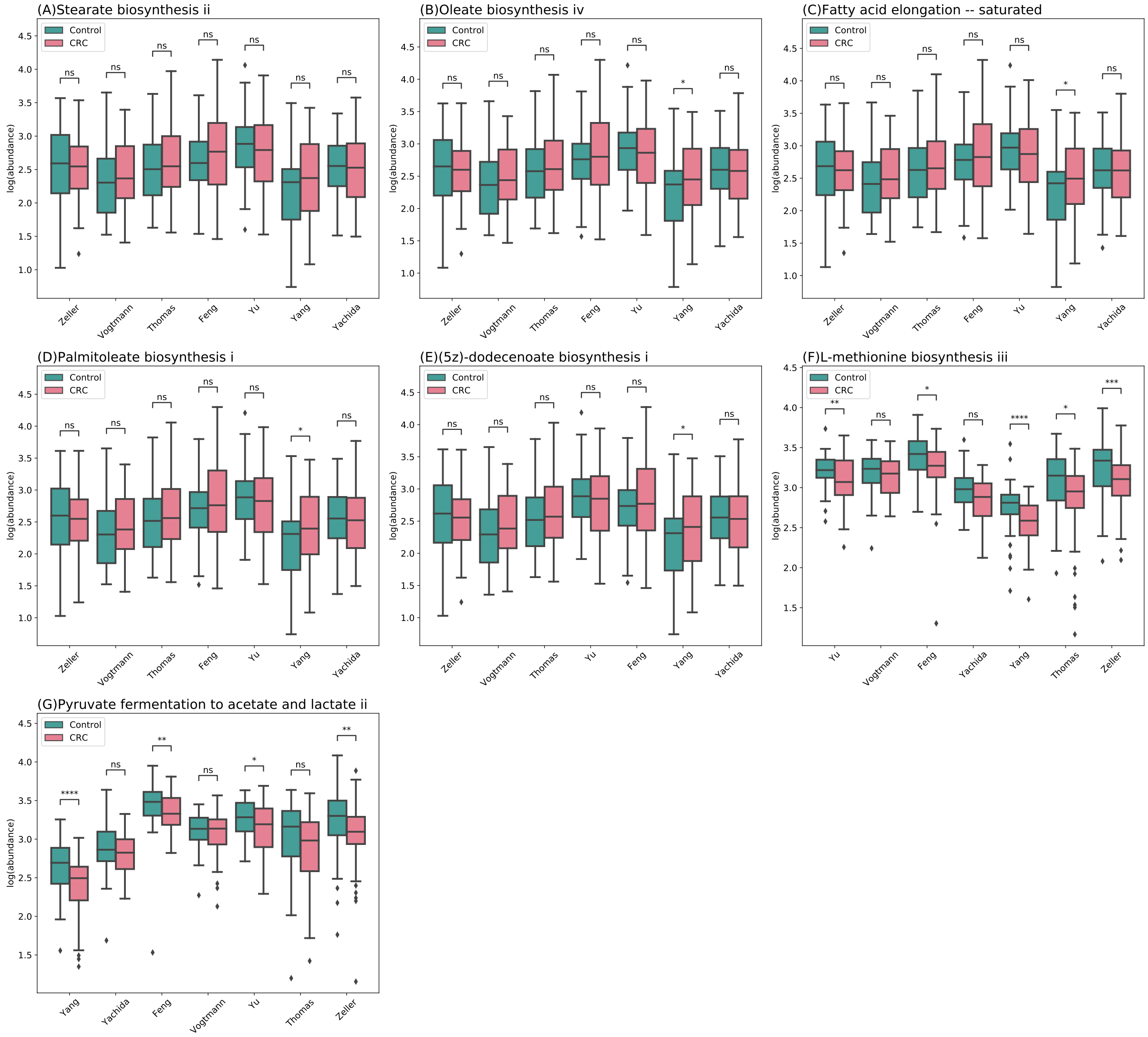


Figure S10: Boxplots showing the log transformed HUMAnN3 pathway abundance of significant pathways within each dataset. P-values were calculated using the two-tailed Wilcoxon rank-sum test. ns:p$>0.05$, *p$<0.05$, **p$<0.01$, ***p$<0.001$, ****p$<0.0001$.

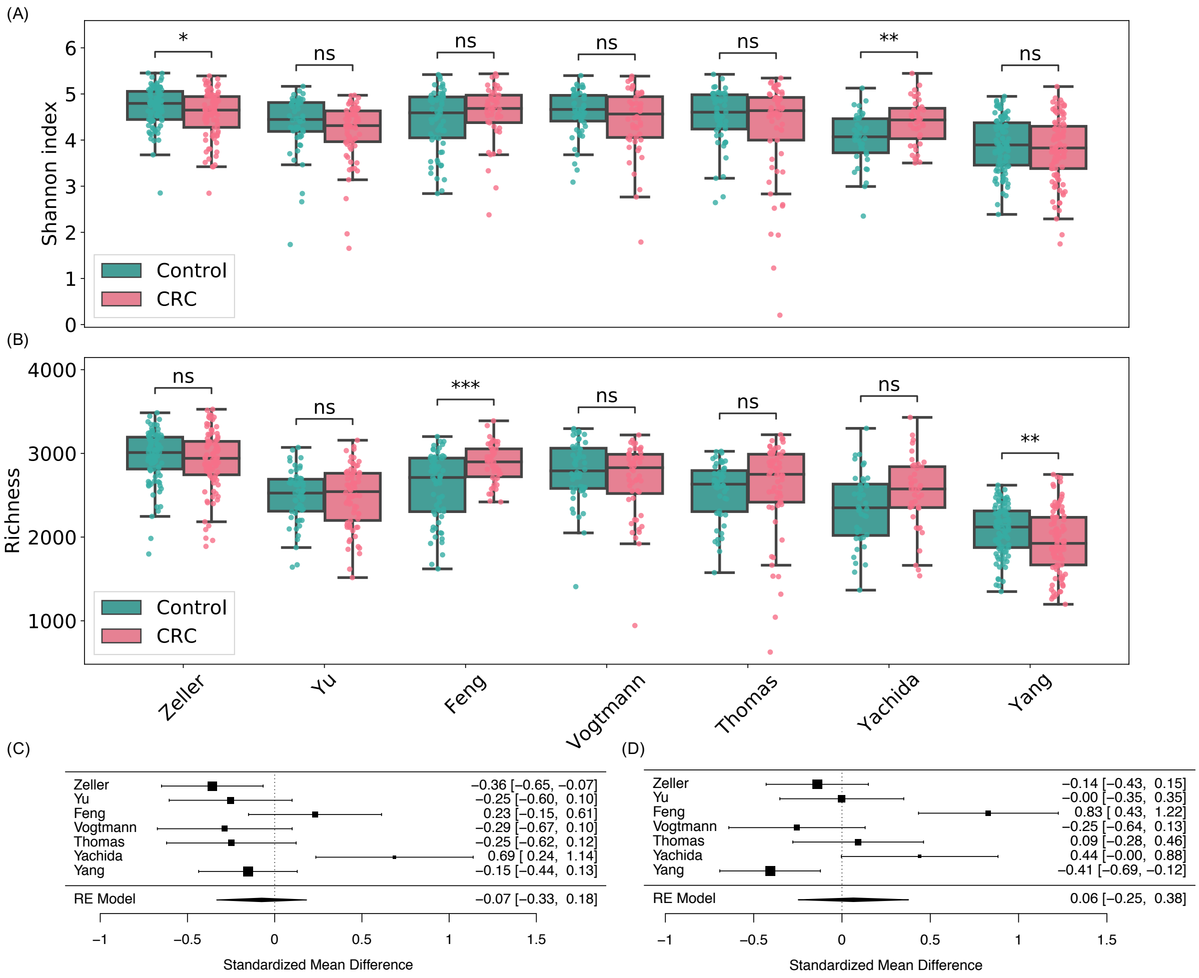


Figure S11: Alpha diversity of bacterial species. (**A**) Boxplots showing bacterial Shannon index. (**B**) Boxplots showing bacterial richness. P-values were calculated using the two-tailed Wilcoxon rank-sum test. ns:p$>$ 0.05, *p$<$ 0.05, **p$<$ 0.01, ***p$<$ 0.001, ****p$<$ 0.0001. (**C**) Forest plot showing effect sizes from a meta-analysis on species-level bacterial diversity (meta-analysis $I^2 = 73.31\%$, Q test p-value $= 0.0029$). (**D**) Forest plot showing effect sizes from a meta-analysis on species-level bacterial richness (meta-analysis $I^2 = 81.82\%$, Q test p-value $< 0.0001$). RE Model: Random effect model.

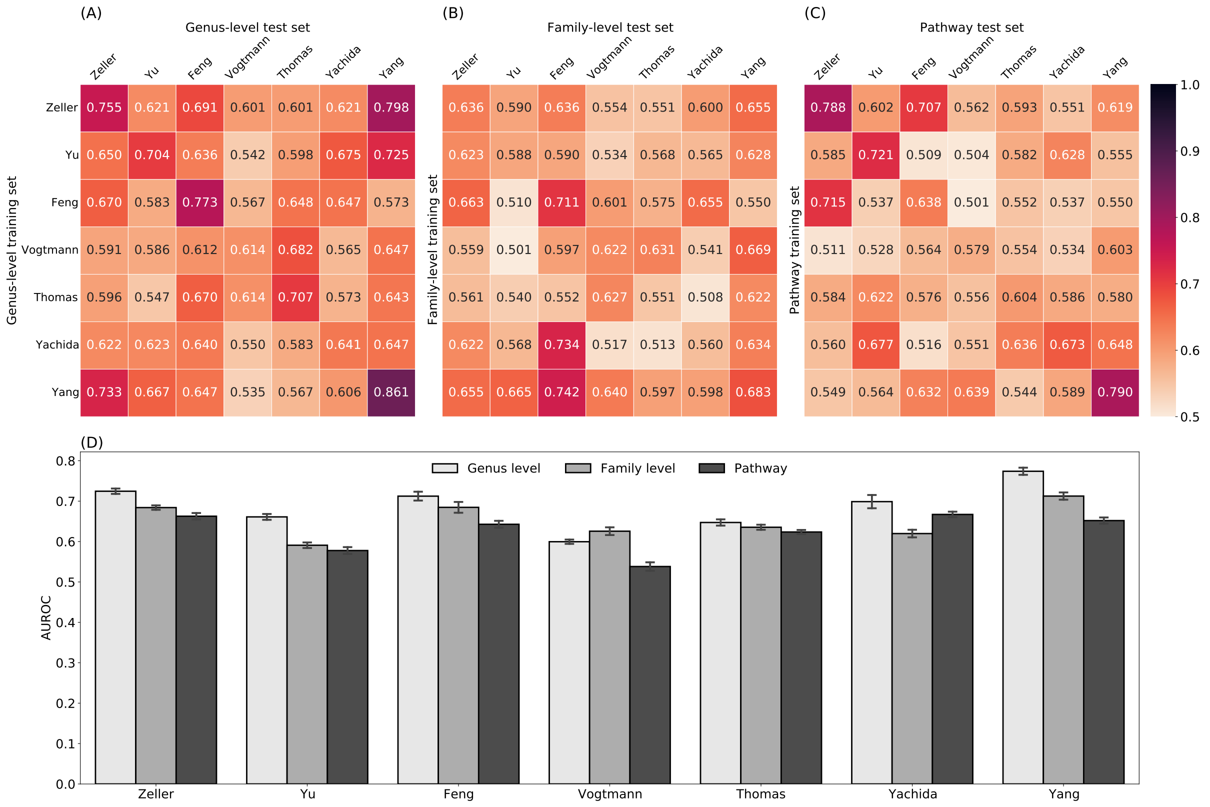


Figure S12: Prediction performances of random forest classifiers based on gut viral abundance. (**A**) Within and cross study AUROC matrix obtained by using genus-level abundance. The diagonal refers to results of cross validation within each dataset. Off-diagonal values refer to prediction results trained on the study on each row and tested on the study on each column. (**B**) Within and cross study AUROC matrix obtained by using family-level abundance. (**C**) Within and cross study AUROC matrix obtained by using pathway abundance. (**D**) LODO results with the x axis indicating the study left out as the validation set and other studies combined as the training set.

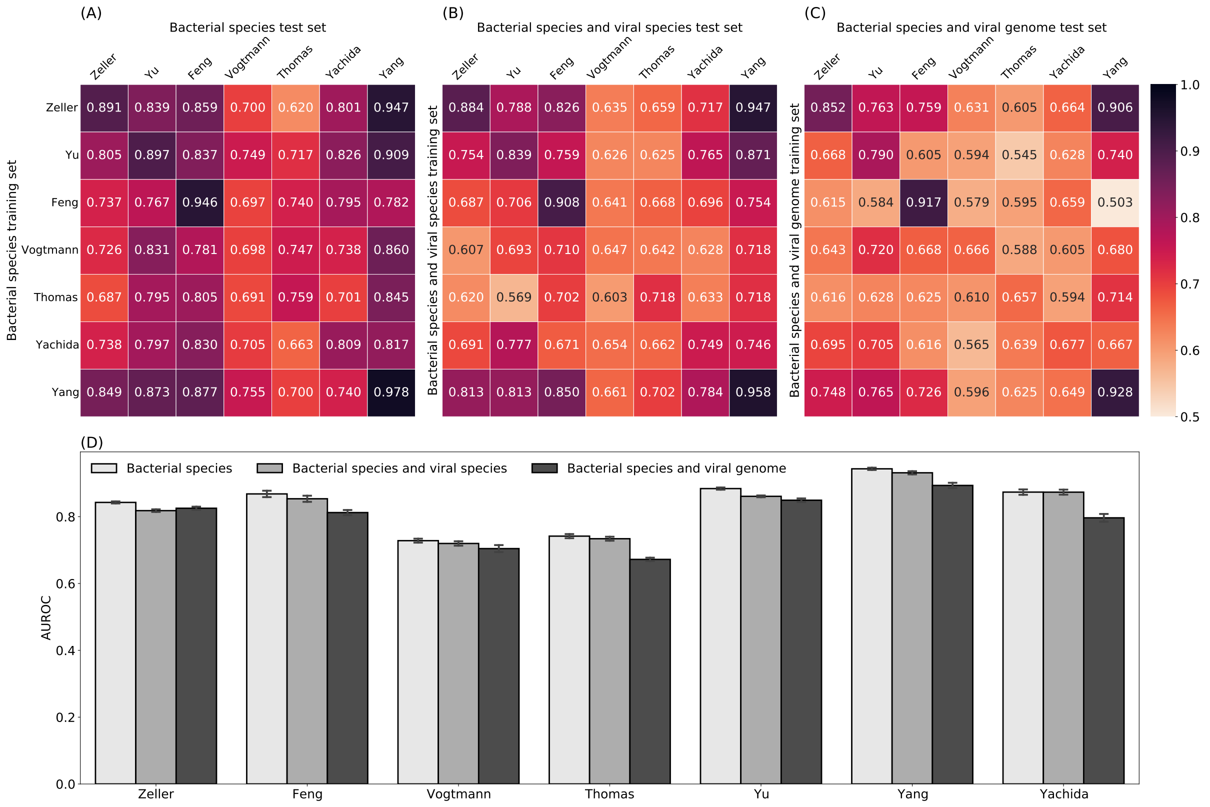


Figure S13: Prediction performances of random forest classifiers based on gut microbial abundance. (**A**) Within and cross study AUROC matrix obtained by using bacterial species-level abundance. The diagonal refers to results of cross validation within each dataset. Off-diagonal values refer to prediction results trained on the study on each row and tested on the study on each column. (**B**) Within and cross study AUROC matrix obtained by using both bacterial and viral species-level abundance profiles. (**C**) Within and cross study AUROC matrix obtained by using both bacterial species-level and viral genome-level abundance profiles. (**D**) LODO results with the x axis indicating the study left out as the validation set and other studies combined as the training set.
